# GaugeFixer: overcoming parameter non-identifiability in models of sequence-function relationships

**DOI:** 10.64898/2025.12.08.693054

**Published:** 2025-12-10

**Authors:** Carlos Martí-Gómez, David M. McCandlish, Justin B. Kinney

**Affiliations:** 1 Simons Center for Quantitative Biology, Cold Spring Harbor Laboratory, 1 Bungtown Rd., Cold Spring Harbor, 11724, New York, United States

## Abstract

Mathematical models that describe sequence-function relationships are widely used in computational biology. A key challenge when interpreting these models is that their parameters are not uniquely determined: many different parameter choices can encode the same sequence-function landscape. These ambiguities, known as “gauge freedoms,” must be resolved before parameter values can be meaningfully interpreted. Resolving gauge freedoms requires imposing mathematical constraints on parameters that remove these degrees of freedom, a procedure called “fixing the gauge.” We recently developed mathematical methods for fixing the gauge of a large class of commonly used models, but the direct computational implementation of these methods is often impractical due to the need for projection matrices whose memory requirements scale quadratically with the number of parameters. Here we introduce GaugeFixer, a Python package that exploits the specific mathematical structure of gauge-fixing projections to achieve linear scaling, thus enabling application to models with millions of parameters. As one application, we analyzed the local structure of peaks in an empirical fitness landscape for translation initiation. GaugeFixer reveals striking similarities, but also fine-scaled variation, in ribosome binding preferences at different positions relative to the start codon, thereby aiding the interpretation of an otherwise unwieldy fitness landscape. GaugeFixer thus fills an unmet need in the computational tools available for biologically interpreting sequence-function relationships.

## Introduction

Computational biology routinely involves the use of models that describe the quantitative relationship between biological sequences (DNA, RNA, or protein) and measurable biological activities [Kinney and McCandlish, 2019]. For example, quantitative models of sequence-function relationships have been used to predict the locations of transcription factor binding sites in promoters and enhancers [Stormo, 2013], the locations of splice sites in pre-mRNA transcripts [Yeo and Burge, 2004], structural contacts between residues in folded proteins [Marks et al., 2012], and the effects of human genetic variation on protein function [Hopf et al., 2017]. One application of growing interest is the modeling of data from high-throughput mutagenesis experiments [Kinney et al., 2010, Mogno et al., 2013, Belliveau et al., 2018, Otwinowski et al., 2018, Faure et al., 2022, Zhou et al., 2022, Tareen et al., 2022, Faure and Lehner, 2024, Martí-Gómez et al., 2025, Zhou et al., 2025].

A common way to mathematically represent sequence-function relationships is to use generalized one-hot models [Posfai et al., 2025b,a]. In these models, each sequence is represented as a set of binary features, each feature indicating the presence or absence of a specific subsequence at a specific set of positions. For example, additive features indicate individual characters (nucleotides or amino acids) at individual positions, pairwise features indicate pairs of characters at pairs of positions, and so on. Each parameter of a generalized one-hot model quantifies the effect that a corresponding sequence feature has. Although generalized one-hot models are linear functions of their parameters, they can represent arbitrarily complex sequence-function landscapes [Posfai et al., 2025b].

A fundamental challenge often arises when one attempts to interpret the parameters of generalized one-hot models: except in unusual cases, these parameters are not uniquely determined by the sequence-function landscape the model describes [Posfai et al., 2025a]. As a consequence, the values of model parameters cannot be uniquely determined by data, even in principle. This non-identifiability occurs because the binary feature vectors that represent sequences do not span the full space in which they live. Consequently, a nontrivial set of parameter vectors will yield identical model predictions. These parameter vectors differ by what are called “gauge freedoms” – directions in parameter space that are orthogonal to all binary feature vectors.

The ambiguity that gauge freedoms produce must be resolved before the values of model parameters can be interpreted, or before the parameters of two different models can be compared. This resolution is achieved by imposing additional mathematical constraints that, together with the predicted landscape, select a unique set of parameter values. This procedure is called “fixing the gauge”. Different choices of constraints yield different gauges, each providing a distinct mathematical representation of the same underlying sequence-function relationship reflecting a distinct interpretation of the parameters.

The importance of gauge freedoms was first recognized in theoretical physics and is very well understood in that context [Jackson and Okun, 2001]. Until recently, however, gauge freedoms in sequence-function relationships had received little attention. A diffuse body of work had discussed specific approaches for handling gauge freedoms in specific modeling contexts, e.g., in additive models for transcription factor binding specificity [Kinney et al., 2007, Stormo, 2011, Rube et al., 2022], pairwise models of protein fitness landscapes [Weigt et al., 2009, Marks et al., 2011, Ekeberg et al., 2013, Barton et al., 2016, Cocco et al., 2018, Haldane and Levy, 2019, Gerardos et al., 2022, Hsu et al., 2022], and all-order interaction models [Zamuner and Rios, 2021, Feinauer et al., 2022, Feinauer and Borgonovo, 2022], and the issue had also arisen implicitly in the literature concerning alternative parameterizations for genetic interactions[Poelwijk et al., 2019, Metzger et al., 2024, Park et al., 2024, Dupic et al., 2024, Faure et al., 2024]. However, a unified understanding of gauge freedoms in sequence-function relationships had yet to be developed.

To address this need, we recently developed a mathematical theory of gauge freedoms in generalized one-hot models [Posfai et al., 2025b,a]. In particular, Posfai et al. [2025b]] introduced a specific family of gauges that can be imposed through the projection of model parameters via matrix multiplication. These gauges have a simple mathematical form and include nearly all of the gauges used in the prior literature. However, carrying out this matrix operation requires storing and manipulating dense projection matrices, the sizes of which scale quadratically with the number of model parameters, and is impractical for models with more than a few thousand parameters.

Here we introduce GaugeFixer, an open-source Python package that overcomes this computational limitation. By exploiting the mathematical structure of generalized one-hot models and the projection matrices described by Posfai et al. [2025b]], GaugeFixer achieves linear scaling in both memory and computation time, enabling rapid gauge fixing for models with millions of parameters. To demonstrate its utility, we apply GaugeFixer here to a fitness landscape for Shine-Dalgarno sequences, represented by a model with nearly 2 million parameters [Kuo et al., 2020, Martí-Gómez et al., 2025]. This analysis illustrates how GaugeFixer can be used to reveal shared sequence requirements across different ribosome binding registers, a phenomenon that is otherwise difficult to discern within such complex sequence-function landscapes.

## Background

### Generalized one-hot models

We consider generalized one-hot models that predict a scalar function *f*(s) for sequences *s* of fixed length *L*. Of particular interest among these models are all-order models, which describe interactions of every order from 0 through L. Such models can represent any possible sequence-function relationship, but the number of parameters they require grows exponentially with sequence length. It is therefore common to use models that include only interactions up to a specified order (*K*-order models) or only between nearby positions (nearest-neighbor, or *K*-adjacent models). These are examples of hierarchical models, a class of generalized one-hot models that remain tractable for longer sequences while still capturing important biological dependencies. See Methods for more information.

### Minimal gauge fixing example

We now illustrate the process of gauge fixing using a minimal example of a sequence-function landscape. Consider sequences of length *L* = 1 built using a two-character alphabet {A, B}. There are only two possible sequences in this example: *s* = A and *s* = B. Consequently, the landscape *f*(*s*) has only two degrees of freedom. The corresponding all-order model, however, has three parameters—a constant-effect parameter (*θ*_0_) and two additive parameters ($\theta_{1}^{A}$ and $\theta_{1}^{B}$) that describe the effect of either possible character at position 1 in the sequence. The all-order model therefore exhibits one gauge freedom. Fixing the gauge involves introducing an additional constraint that removes this degree of freedom, leading each valid choice of parameters to produce a unique sequence-function landscape.

**Figure 1:**
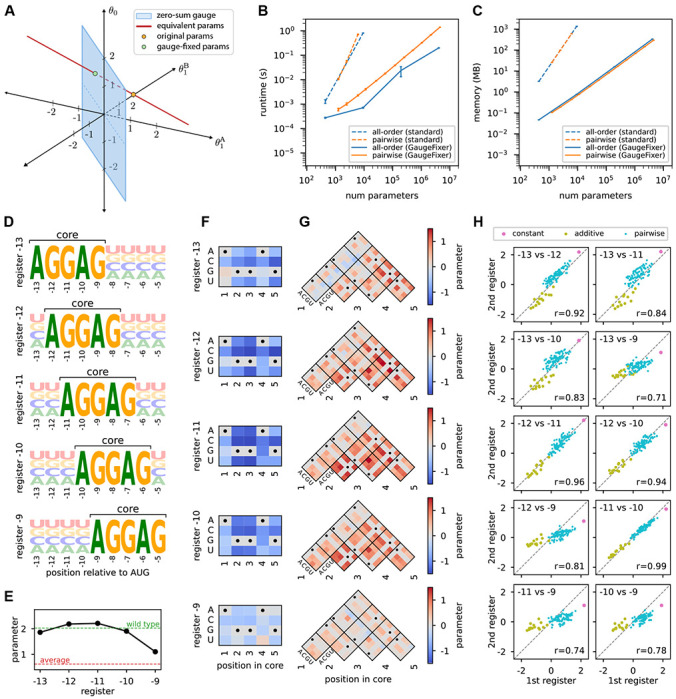
Illustration of gauge-fixing and demonstration of GaugeFixer. (A) Minimal example of gauge fixing. Shown is the space of all possible three-dimensional parameter vectors, $\vec{\theta }=[\theta_{0},\theta_{1}^{A},\theta_{1}^{B}]$, for the all-order interaction model on sequences of length *L* = 1 built from the alphabet {*A, B*}. Orange dot: initial choice of $\vec{\theta }=\left[0,0,2\right]$, which yields *f*(*A*) = 0, *f*(*B*) = 2. Red line: parameter vectors equivalent to $\vec{\theta }$. Blue plane: parameter vectors in the zero-sum gauge, i.e., that satisfy $\theta_{1}^{A}+\theta_{1}^{B}=0$. Green dot: gauge-fixed parameters ${\vec{\theta }}_{\text{fixed}}=[1,-1,1]$. (B,C) Algorithm performance when fixing the gauge of all-order and pairwise protein models with increasing number of parameters. Shown are the runtime (B) and peak memory (C) requirements for GaugeFixer and for standard matrix multiplication. Error bars represent the standard deviation in runtime and memory usage over 10 projections of parameter vectors drawn i.i.d. from a normal distribution. (D) Sequence logos showing the probability distributions *π* corresponding to different peaks in the Shine-Dalgarno fitness landscape measured by Kuo et al. [2020] and modeled by Martí-Gómez et al. [2025]. Each distribution has a fixed AGGAG motif at a specific register relative to the start codon. (E,F,G) Gauge-fixed constant (E), additive (F), and pairwise (G) parameters in the hierarchical gauge associated with each register. Horizontal dashed lines in (E) represent the average phenotype across all possible sequences (red) and the phenotype of the wild-type sequence AAGGAGGUG (green), which occurs in the 5’UTR of the *dmsC* gene of *Escherichia coli*. (H) Scatterplots comparing the values of gauge-fixed parameters observed in the core region at different registers.

Figure 1A illustrates this example. The orange dot shows one choice of parameter vector, $\vec{\theta }=[\theta_{0},\theta_{1}^{A},\theta_{1}^{B}]=[0,0,2]$. This choice yields the following sequence-function landscape: $f(A)=\theta_{0}+\theta_{1}^{A}=0,f(B)=\theta_{0}+\theta_{1}^{B}=2$. There are, however, an infinite number of equivalent parameter vectors that yield this same landscape, namely vectors of the form [*z*, −*z*, 2 – *z*] where *z* is any real number (red line). Fixing the gauge involves specifying an additional constraint that selects a single point on this line. The blue plane in Figure 1A represents one such constraint: the zero-sum gauge, which requires that the additive parameters sum to zero at each position ($\theta_{1}^{A}+\theta_{1}^{B}=0$). The resulting gauge-fixed parameters (orange dot) lie at the intersection of the line of equivalent parameters with the zero-sum plane (${\vec{\theta }}_{\text{fixed}}=\left[1,-1,1\right]$; green dot). Note that no choice of parameters in the zero-sum gauge other than ${\vec{\theta }}_{\text{fixed}}$ will yield the specific landscape *f*(*A*) = 0, *f*(*B*) = 2.

### Families of gauges

In previous work [Posfai et al., 2025b], we introduced a family of gauges for the all-order interaction model. These gauges are parameterized by two quantities: a non-negative number *λ* and a probability distribution *π* over sequences. *λ* controls how much explanatory power is allocated across interaction orders, while *π* specifies the distribution of sequences against which parameter effects are expressed. This *λ, π* family of gauges has multiple attractive properties. In particular, it encompasses the most commonly used gauges in the literature including the trivial gauge, the euclidean gauge, the equitable gauge, the zero-sum gauge, and the wild-type gauge. See Posfai et al. [2025b] for details.

A particularly useful subset of gauges in this family are the hierarchical gauges, which are obtained in the limit where *λ* approaches infinity. In these gauges, lower-order terms explain as much of the landscape’s variance as possible, while higher-order interaction terms capture only residual variation that cannot be attributed to lower-order effects. Unlike other gauges in the *λ, π* family, hierarchical gauges can be applied to all hierarchical models. The values of model parameters in hierarchical gauges also have a natural interpretation: each parameter represents the average effect of introducing specific characters at specific positions, compared to the effect expected from lower-order terms, when sequences are drawn from the distribution *π*. By choosing different distributions *π*, one can observe how sequence-function relationships vary across different regions of sequence space. For example, a uniform distribution yields global averages, whereas distributions concentrated near particular sequences reveal local landscape structure.

## Results

### The GaugeFixer algorithm

Fixing the gauge requires projecting parameter vectors onto lower-dimensional subspaces. The projection matrices for gauges in the *λ, π* family have a special structure: they can be written as Kronecker products of *L* much smaller matrices, one matrix for each sequence position [Posfai et al., 2025b]. GaugeFixer exploits this factorization to compute projections without ever constructing the full projection matrix. This is possible because matrix-vector products involving a Kronecker product of *L* matrices can be computed iteratively, applying one small matrix at a time to successive dimensions of the parameter array [Martí-Gómez et al., 2025]. This reduces both the memory requirements and computation time from O(*M*^2^) to O(*M*), where *M* is the number of model parameters. For hierarchical models more generally, GaugeFixer decomposes the model into a sum of all-order models restricted to subsets of positions, applies the efficient projection algorithm to each, and sums the results together. See Methods for details.

Figures 1B,C show the performance advantage of this approach. Compared to direct matrix multiplication, GaugeFixer achieves orders of magnitude improvement in both runtime and memory usage. This makes it possible to gauge-fix models with millions of parameters in just a few seconds on a standard laptop computer. By contrast, gauge-fixing becomes impractical beyond about 10^4^ parameters when using explicit projection matrices.

### Analysis of the Shine-Dalgarno fitness landscape

To illustrate the utility of GaugeFixer, we analyzed the fitness landscape of the Shine-Dalgarno (SD) sequence, a motif in bacterial messenger RNA that facilitates translation initiation by base-pairing with the 3’ tail of the 16S ribosomal RNA [Shine and Dalgarno, 1975]. Kuo et al. [2020] previously measured the translational activity of nearly every possible 9-nucleotide RNA sequence. Subsequent modeling efforts inferred a sequence-function landscape from this data, corresponding to an all-order model with 5^9^ = 1, 953, 125 parameters [Zhou et al., 2022, Martí-Gómez et al., 2025]. This landscape contains multiple fitness peaks corresponding to the canonical AGGAG motif positioned in different registers relative to the start codon.

We used GaugeFixer to quantitatively characterize the local structure around fitness peaks in the landscape inferred by Martí-Gómez et al. [2025]. For each peak we defined a distribution *π* that fixes the AGGAG motif in the corresponding register while randomly drawing characters at the remaining positions (Figure 1D). We then imposed the hierarchical gauge corresponding to each distribution *π* and examined the resulting parameter values. In this gauge, the constant, additive, and pairwise parameters can be respectively interpreted as the average phenotype, average single-nucleotide effect, and average epistatic effect observed when mutations are introduced into sequences randomly drawn from *π*. We emphasize that these different parameter values, corresponding to different gauges, are simply different representations of the same model.

The constant term, *θ*_0_, represents the mean fitness of sequences with AGGAG in the specified register. We find that this value is highest for registers −12 and −11, consistent with known optimal spacing requirements for translation initiation (Figure 1E). In register −9, by contrast, the mean fitness is much lower, indicating markedly lower translation on average.

The additive parameters in the core region, shown in Figure 1F, reveal how individual nucleotide mutations away from the AGGAG motif affect translational efficiency in each register. As expected, these mutations are overwhelmingly deleterious. The effects are also remarkably consistent across registers, though some differences emerge near the boundaries.

The pairwise interaction parameters in the core region, shown in Figure 1G, capture the effects of mutating pairs of nucleotides within the AGGAG motif beyond what additive effects alone predict. These interactions are also remarkably consistent across registers. Predominantly positive values are observed, indicating that combinations of mutations tend to be less deleterious than expected from their individual effects, one hallmark of global epistasis [Otwinowski et al., 2018, Kinney and McCandlish, 2019].

Finally, we compared the constant, additive, and pairwise parameters in the core region across registers (Figure 1H). The results show that neighboring registers tend to have more similar parameters, while more distant registers show greater divergence. This smooth variation suggests that ribosomal binding preferences change gradually as a function of distance from the start codon.

## Discussion

Here we introduced GaugeFixer, a Python package that implements a computationally efficient algorithm for removing gauge freedoms from generalized one-hot models of sequence-function relationships. By exploiting the Kronecker factorization of projection operators and the specific mathematical structure of the models, GaugeFixer dramatically reduces runtime and memory requirements, thus enabling rapid gauge fixing for models with millions of parameters.

We wish to emphasize that gauge fixing is fundamentally different from parameter inference. Both procedures are an essential part of modeling sequence-function relationships, but they serve orthogonal purposes. Inference is the process of identifying parameters that make a model’s predictions best fit one’s data, but without regard to parameter interpretation. Gauge-fixing, by contrast, alters model parameters in ways that have no effect on model predictions, but which are important for the interpretation of those parameters. GaugeFixer makes this distinction explicit by providing utilities to convert precomputed parameter vectors into any chosen gauge. A related but more subtle point is that some regularization schemes and/or prior distributions used during inference yield parameters in specific gauges [Posfai et al., 2025b, Petti et al., 2025]. This does not, however, eliminate the need for post-inference gauge fixing. On the contrary, the most common/effective regularization methods often produce parameters in gauges that are not consistent with how one wishes to interpret those parameters.

Although GaugeFixer is designed to work with specific classes of linear models (all-order models and hierarchical models), it can also be applied to nonlinear and nonparametric models, such as neural networks or Gaussian processes. This is done by representing (or approximating) the model-predicted landscape using an all-order (or hierarchical) model, then applying GaugeFixer to the parameters of that model. Here we demonstrated this capability by analyzing a Shine-Dalgarno fitness landscape modeled using Gaussian process regression [Zhou et al., 2022, Martí-Gómez et al., 2025]. Martí-Gómez et al. [2025] recently described gpmap-tools, a Python package that enables such Gaussian process modeling when sequences are short enough to enumerate all possible interaction terms. GaugeFixer complements this functionality by enabling post-hoc computation of gauge-fixed parameters for any of these models. We note, however, that recent theoretical results suggest the possibility of directly computing the posterior distribution over specific gauge-fixed parameters using Gaussian processes defined over sequences of arbitrary length, thus bypassing the need to first construct explicit all-order models [Petti et al., 2025].

Different gauges have different advantages when interpreting model parameters. For instance, the influence of global epistasis is more evident when parameters are represented in the wild-type gauge, whereas using additive models to illustrate the specificities of transcription factors, or computing contact maps in pairwise interaction models, typically requires the models to be in the zero-sum gauge [Kinney et al., 2007, Stormo, 2011, Rube et al., 2022, Marks et al., 2011, Ekeberg et al., 2013]. Gauge-fixing is conceptually related to other techniques for interpretation of neural network models such as global importance analysis [Koo et al., 2021], which quantifies the average effect of placing a specific sequence pattern, e.g., a transcription factor binding site, within random genetic backgrounds. While this method has the advantage that it can be applied to any model, gauge-fixing enables exact computation, not only of the average effect of a sequence pattern over random genetic backgrounds, but also the expected effects of mutations and their combinations. By doing so, gauge-fixing facilitates the investigation of how mutational effects and their interactions change across different regions of sequence space, providing an intuitive way to interpret the higher-order interactions that make them different.

GaugeFixer thus provides a high-performance software library for fixing the gauge of commonly used sequence-to-function models, filling an important gap in the computational tools available for interpreting sequence-function relationships.

## Methods

### Generalized one-hot models

We consider scalar-valued sequence-function relationships *f*(*s*) where *s* is a sequence of fixed length *L* built from an alphabet $𝒜=\{c_{1},c_{2},\ldots ,c_{\alpha }\}$ comprising *α* distinct characters.^1^ Let *S* = {1, 2, … , *L*} denote the set of positions within *s*. Linear models of sequence-function relationships have the form

(1) $$f(s)=\vec{\theta }\cdot \vec{x}(s),$$

where $\vec{\theta }$ is a vector of model parameters and $\vec{x}(s)$ is a vector of sequence-dependent features.

This work focuses on a specific class of linear models, the generalized one-hot models described by Posfai et al. [2025b,a]. In these models, the sequence-dependent features are binary indicator functions over subsequences. Formally, each feature is given by

(2) $$x_{U}^{u}(s)=\{\begin{array}{ll} 1 & \text{if}\;s[U]=u\\ 0 & \text{otherwise} \end{array},$$

where *U* ⊆ *S* is a subset of positions, *s*[*U*] indicates the subsequence of *s* that occurs at these positions, and *u* is a sequence of length |*U*| (i.e., $u\in {𝒜}^{\left|U\right|}$).^2^ Generalized one-hot models also require that $\vec{x}(s)$ be equivariant under position-specific character permutations [Posfai et al., 2025a]. This means that if $x_{U}^{u}$ occurs in $\vec{x}$ for some *U* and subsequence *u*, so must $x_{U}^{u^{\prime }}$ for every possible subsequence *u*′ of size |*U*|. The set of features in a generalized one-hot model is therefore defined by an alphabet 𝒜 and a collection of position sets *V* = {*U*_1_, *U*_2_, … , *U_n_*} via

(3) $$f(s)=\underset{U\in V}{\sum }\underset{u\in {𝒜}^{\left|U\right|}}{\sum }\theta_{U}^{u}x_{U}^{u}(s).$$

### All-order models and hierarchical models

All-order models are generalized one-hot models that include features for all possible subsequences at all possible sets of positions. This means that *V* includes every possible position set *U* ⊆ *S*. *V* is therefore the powerset of *S*, denoted V=𝒫(S). Because the subsets of *S* fully define *V*, we say that *S* is the generating position set of *V*.

Hierarchical models are more general than all-order models. Each hierarchical model is defined by one or more generating position sets *S*′ ⊆ *S*, the subsets of which together comprise *V* . Formally, if $W=\left\{S_{1}^{\prime },S_{2}^{\prime },\ldots ,S_{m}^{\prime }\right\}$ denotes the set of generating position sets, then $V=\cup_{S^{\prime }\in W}𝒫(S^{\prime })$ is the union of the powersets of these generating position sets. Common examples of hierarchical models include additive models (defined by all position sets of size 0 and 1), pairwise models (all position sets up to size 2), nearest-neighbor models (same as pairwise but with size-2 position sets restricted to adjacent positions), *K*-order models (all position sets up to size *K*), and *K*-adjacent models (all position sets up to size *K* but restricted *K* adjacent positions).^3^

### Gauge-fixing projection matrices

The *λ, π* family is a set of gauges for the all-order interaction model. It is parameterized by a non-negative number *λ* and a probability distribution *π* over sequences that factorizes over positions. This family includes many commonly used gauges. In particular, the hierarchical gauges correspond to setting *λ* = ∞ and generalizes two commonly used gauges. The zero-sum gauge, commonly used when modeling transcription factor binding to DNA, is the hierarchical gauge that results from *π* being uniform. Similarly, defining *π* to be an indicator function for a specific sequence yields the wild-type gauge, which is commonly used in studies of protein fitness landscapes.

Fixing the gauge of all-order models is accomplished by linear projection. In this case there are *M* = (*α* + 1)^*L*^ parameters. Given an *M*-dimensional vector $\vec{\theta }$ of initial parameter values, one can obtain the gauge-fixed parameters ${\vec{\theta }}_{\text{fixed}}$ via multiplication with an appropriate *M* × *M* projection matrix *P* (which depends on *λ* and *π*):

(4) $${\vec{\theta }}_{\text{fixed}}=P\vec{\theta }.$$

Importantly, the projection matrix can be written as a Kronecker product of position-specific matrices:

(5) $$P=P_{1}⊗P_{2}⊗\cdots ⊗P_{L},$$

where each *P_l_* is the (*α* + 1) × (*α* + 1) matrix

(6) $$P_{l}=\left(\begin{array}{ccccc} \eta & \pi_{l}^{c_{1}}\eta & \pi_{l}^{c_{2}}\eta & \cdots & \pi_{l}^{c_{\alpha }}\eta \\ 1-\eta & 1-\pi_{l}^{c_{1}}\eta & -\pi_{l}^{c_{2}}\eta & \cdots & -\pi_{l}^{c_{\alpha }}\eta \\ 1-\eta & -\pi_{l}^{c_{1}}\eta & 1-\pi_{l}^{c_{2}}\eta & \cdots & -\pi_{l}^{c_{\alpha }}\eta \\ \vdots & \vdots & \vdots & \ddots & \vdots \\ 1-\eta & -\pi_{l}^{c_{1}}\eta & -\pi_{l}^{c_{2}}\eta & \cdots & 1-\pi_{l}^{c_{\alpha }}\eta \end{array}\right),$$

$\pi_{l}^{c}$ is the probability of character c occurring at position *l*, and *η* = *λ*/(1 + *λ*). See Posfai et al. [2025b] for details.

Although the *λ, π* gauges are defined in the context of all-order models, the specific class of hierarchical gauges can also be applied to the more general class of hierarchical models. This is done by treating each hierarchical model on sequences of length *L* as an all-order model on sequences of the same length that has unused parameters set to zero. This works because hierarchical gauges preserve these sets of zero-valued parameters. Other *λ, π* gauges do not preserve these zero-valued parameters, and therefore are restricted in their application to all-order models only.

### Efficient gauge fixing via Kronecker factorization

To compute $P\vec{\theta }$, we first reshape $\vec{\theta }$ to be an *L*-dimensional tensor having size (*α* + 1) along each dimension. The expression in Eq. 4 then becomes

(7) $${\left[{\vec{\theta }}_{\text{fixed}}\right]}_{i_{1}\cdots i_{L}}=\underset{j_{1}\cdots j_{L}}{\sum }{\left[P_{1}\right]}_{i_{1}j_{1}}\cdots {\left[P_{L}\right]}_{i_{L}j_{L}}{\left[\vec{\theta }\right]}_{j_{1}\cdots j_{L}}.$$

We compute Eq. 7 iteratively: first we define ${\vec{\theta }}^{\left(0\right)}=\vec{\theta }$; then for *l* = 1, 2, ⋯ , *L* we compute

(8) $${\vec{\theta }}_{i_{1}\cdots i_{L}}^{\left(l\right)}=\underset{j_{l}}{\sum }{\left[P_{l}\right]}_{i_{l}j_{l}}{\left[{\vec{\theta }}^{\left(l-1\right)}\right]}_{i_{1}\cdots i_{l-1}j_{l}i_{l+1}\cdots i_{L},}$$

which ultimately yields ${\vec{\theta }}_{\text{fixed}}={\vec{\theta }}^{\left(L\right)}$. This computation is formalized in Algorithm 1, which uses a single intermediate parameter vector $\vec{\psi }$ to avoid storing all ${\vec{\theta }}^{\left(l\right)}$ simultaneously. Algorithm 1 has a computational complexity of O(*L*(*α* + 1)*M*). This compares favorably to the computational complexity of direct matrix multiplication of $\vec{\theta }$ by *P*, which is O(*M*^2^). The memory requirements of Algorithm 1 also scale linearly with *M*, as opposed to quadratically with *M* for direct matrix multiplication.

**Algorithm 1:** Fixing the gauge of all-order models

**Require:** $\text{λ},\text{π},{\vec{\theta }}_{\text{init}}$	
1: $\vec{\psi }\leftarrow \text{reshape}\left({\vec{\theta }}_{\text{init}}\right)$	
2: **for** *l* = 1 to *L* **do**	
3: $P_{l}\leftarrow \text{ProjectionMatrix}\left({\text{λ,π}}_{l}\right)$	▷ Equation 6
4: $\vec{\psi }\leftarrow \text{tensordot}\left(P_{l},\vec{\psi },l\right)$	▷ Equation 8
5: **end for**	
6: ${\vec{\theta }}_{\text{fixed}}\leftarrow \text{reshape}\left(\vec{\psi }\right)$	
**return** ${\vec{\theta }}_{\text{fixed}}$	

### Hierarchical model decomposition

To project a hierarchical model into a hierarchical gauge, we leverage the fact that the parameters of the model can be expressed as a sum of the parameters of multiple all-order models, each restricted to one of the generating position sets. Let $\vec{\theta }$ be the parameter vector of the hierarchical model expressed as an (α+1)*^L^*-dimensional all-order parameter vector (with unused parameters set to zero), and let *W* denote the set of generating position sets. We first decompose $\vec{\theta }$ as

(9) $$\vec{\theta }=\underset{S^{\prime }\in W}{\sum }{\vec{\theta }}_{S^{\prime }}$$

where each ${\vec{\theta }}_{S^{\prime }}$ is an all-order parameter vector for the position set *S*′, i.e., has nonzero parameters $\theta_{U}^{u}$ only for *U* ⊆ *S*′. We note that there are multiple ways to carry out this decomposition, all of which yield the same gauge-fixed parameters. The gauge-fixed parameters are then computed as

(10) $${\vec{\theta }}_{\text{fixed}}=\underset{S^{\prime }\in W}{\sum }P{\vec{\theta }}_{S^{\prime }}$$

Importantly, each term on the right-hand side can be computed using only the Kronecker factors *P_l_* corresponding to positions *l* ∈ *S*′. Thus, there is never any need to represent the full (*α* + 1)*^L^*-dimensional vectors $\vec{\theta }$, ${\vec{\theta }}_{S^{\prime }}$, or ${\vec{\theta }}_{\text{fixed}}$.

Algorithm 2 formalizes this procedure. Here, $\vec{\theta }[S^{\prime }]$ indicates an (*α* + 1)^|*S*′|^-dimensional vector composed only of the elements $\theta_{U}^{u}$ for which *U* ⊆ *S*′, and *π* [S^′^] represents the marginal distribution *π* over subsequences on *S*′. Note that the iterative form of this algorithm allows us to avoid simultaneously storing the nonzero elements of all ${\vec{\theta }}_{S^{\prime }}$ vectors.

**Algorithm 2:** Fixing the gauge of hierarchical models.

**Require:** $\text{π},{\vec{\theta }}_{\text{init}},W$	
1: ${\vec{\theta }}_{\text{fixed}}\leftarrow \vec{0}$	
2: ${\vec{\theta }}_{\text{tmp}}\leftarrow {\vec{\theta }}_{\text{init}}$	
3: **for** *S*′ in *W* **do**	
4: $\vec{\omega }\leftarrow {\vec{\theta }}_{\text{tmp}}\left[S^{\prime }\right]$	▷ Note: $\vec{\omega }={\vec{\theta }}_{S^{\prime }}\left[S^{\prime }\right]$
5: ${\vec{\omega }}_{\text{fixed}}\leftarrow \text{FixAllOrders}\left(\infty ,\pi ,\left[S^{\prime }\right],\vec{\omega }\right)$	▷ Algorithm 1
6: ${\vec{\theta }}_{\text{fixed}}\left[S^{\prime }\right]\leftarrow {\vec{\theta }}_{\text{fixed}}\left[S^{\prime }\right]+{\vec{\omega }}_{\text{fixed}}$	
7: ${\vec{\theta }}_{\text{tmp}}\left[S^{\prime }\right]\leftarrow \vec{\theta }$	
8: **end for**	
**return** ${\vec{\theta }}_{\text{fixed}}$	

## Availability and implementation:

GaugeFixer is compatible with Python ≥ 3.10 and can be installed using the pip package. Documentation is provided at http://gaugefixer.readthedocs.io. Source code is available at http://github.com/jbkinney/gaugefixer, as are the scripts used to carry out the analyses presented here.

## Footnotes

1 GaugeFixer actually supports sequences built using different alphabets at different positions, with no need for these alphabets to be of the same size. The formulas in this section generalize in a straightforward way to accommodate this more general use case.

2 Sets of positions are called “orbits” in [Posfai et al., 2025b,a]

3 Posfai et al. [2025a] adopted a different definition of *K*-adjacent models, one that makes these models non-hierarchical. We favor the present definition, which is hierarchical and thus amenable to gauge fixing using hierarchical gauges.
