# GaugeFixer: overcoming parameter non-identifiability in models of sequence-function relationships

**DOI:** 10.64898/2025.12.08.693054

**Published:** 2026-02-25

**Authors:** Carlos Martí-Gómez, David M. McCandlish, Justin B. Kinney

**Affiliations:** 1 Simons Center for Quantitative Biology, Cold Spring Harbor Laboratory, 1 Bungtown Rd., Cold Spring Harbor, 11724, New York, United States

## Abstract

Mathematical models that describe sequence-function relationships are widely used in computational biology. A key challenge when interpreting these models is that their parameters are not uniquely determined: many different parameter choices can encode the same sequence-function landscape. These ambiguities, known as “gauge freedoms,” must be resolved before parameter values can be meaningfully interpreted. Resolving gauge freedoms requires imposing mathematical constraints on parameters that remove these degrees of freedom, a procedure called “fixing the gauge.” We recently developed mathematical methods for fixing the gauge of a large class of commonly used models, but the direct computational implementation of these methods is often impractical due to the need for projection matrices whose memory requirements scale quadratically with the number of parameters. Here we introduce GaugeFixer, a Python package that exploits the specific mathematical structure of gauge-fixing projections to achieve linear scaling, thus enabling application to models with millions of parameters. To demonstrate GaugeFixer, we analyze the local structure of peaks in an empirical fitness landscape for translation initiation. GaugeFixer reveals striking similarities, but also fine-scaled variation, in ribosome binding preferences at different positions relative to the start codon, thereby facilitating the interpretation of an otherwise unwieldy fitness landscape. GaugeFixer thus fills an unmet need in the computational tools available for biologically interpreting sequence-function relationships.

## Introduction

Computational biology routinely involves the use of models that describe the quantitative relationship between biological sequences (DNA, RNA, or protein) and their biological activities [Kinney and McCandlish, 2019]. For example, quantitative models of sequence-function relationships have been used to predict the locations of transcription factor binding sites in promoters and enhancers [Stormo, 2013], the locations of splice sites in pre-mRNA transcripts [Yeo and Burge, 2004], structural contacts between residues in folded proteins [Marks et al., 2012], and the effects of human genetic variation on protein function [Hopf et al., 2017]. One application of growing interest is the modeling of data from high-throughput mutagenesis experiments [Kinney et al., 2010, Mogno et al., 2013, Belliveau et al., 2018, Otwinowski et al., 2018, Faure et al., 2022, Zhou et al., 2022, Tareen et al., 2022, Faure and Lehner, 2024,Martí-Gómez et al., 2026, Zhou et al., 2025].

A common way to mathematically represent sequence-function relationships is through the use of generalized one-hot models. In these models, each sequence is represented as a set of binary features indicating the presence or absence of specific subsequences at specific sets of positions, and each feature has a corresponding parameter that quantifies its effect. An important challenge when interpreting these parameters is that they are not uniquely determined by the fitness landscape they describe, as different choices of model parameters can yield the same landscape. These extra degrees of freedom in parameter space are called “gauge freedoms” (see Supplementary Information (SI), Secs. 1–2, for formal definitions). Before the numerical values of model parameters can be meaningfully interpreted, gauge freedoms must first be removed by a process called “fixing the gauge” [Posfai et al., 2025b,a].

Gauge freedoms have long played a central role in theoretical physics [Jackson and Okun, 2001], but they have received remarkably little attention in the context of sequence-function relationships. A scattered body of work has described technical methods for handling gauge freedoms in specific sequence-to-function modeling applications [Kinney et al., 2007, Stormo, 2011, Rube et al., 2022, Weigt et al., 2009, Marks et al., 2011, Ekeberg et al., 2013,Barton et al., 2016, Cocco et al., 2018, Haldane and Levy, 2019, Gerardos et al., 2022, Hsu et al., 2022, Zamuner and Rios, 2021, Feinauer et al., 2022, Feinauer and Borgonovo, 2022]. Other work has explored ways of eliminating these freedoms through alternative parameterizations of genetic interactions [Poelwijk et al., 2019, Metzger et al., 2024, Park et al., 2024b, Dupic et al., 2024, Park et al., 2024a, Faure et al., 2024]. However, a unified approach for handling gauge freedoms had been missing. To address this need, we recently developed a mathematical theory of gauge freedoms for generalized one-hot models [Posfai et al., 2025a]; see also [Posfai et al., 2025b]. This work showed that a family of gauges that encompasses nearly all of the gauges used in the prior literature can be imposed by multiplying parameter vectors by an appropriate projection matrix. Despite its simplicity, directly implementing this gauge-fixing procedure is often impractical for models with more than a few thousand parameters due to the required projection matrices being very large (and not sparse).

Here we introduce GaugeFixer, an open-source Python package for fixing the gauge of generalized one-hot models of sequence-function relationships using the mathematical methods developed by Posfai et al. [2025a]. By exploiting the mathematical structure of generalized one-hot models and their corresponding gauge-fixing projection matrices, GaugeFixer achieves linear scaling in both memory and computation time, enabling the application to models with millions of parameters. To demonstrate its utility, we apply GaugeFixer here to a fitness landscape for Shine-Dalgarno sequences described by a model with nearly 2 million parameters [Kuo et al., 2020,Martí-Gómez et al., 2026]. The results illustrate how GaugeFixer can be used to reveal phenomena that are otherwise difficult to discern within complex sequence-function landscapes.

## Background

### Generalized one-hot models

We consider generalized one-hot models that predict a scalar function f(s) for sequences s of fixed length L. Of particular interest among these models are all-order models, which describe interactions of every order from 0 through L. Such models can represent any possible sequence-function relationship, but the number of parameters they require grows exponentially with sequence length. It is therefore common to use models that include only interactions up to a specified order (*K*-order models) or only between nearby positions (nearest-neighbor or *K*-adjacent models). These are examples of hierarchical models, a class of generalized one-hot models that remain tractable for longer sequences while still capturing important biological dependencies (SI Sec. 2).

### Minimal gauge fixing example

**Figure 1:**
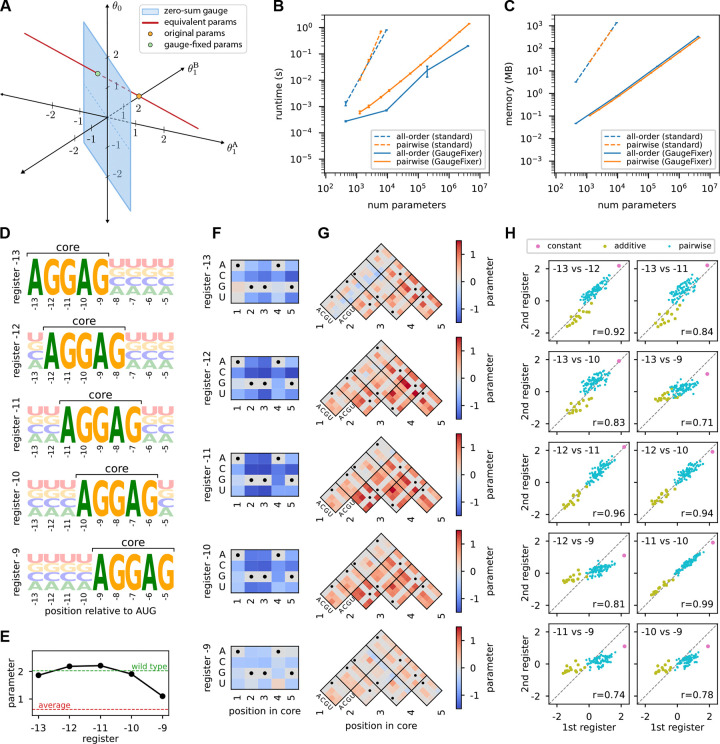
Illustration of gauge-fixing and demonstration of GaugeFixer. (A) Minimal example of gauge fixing. Shown is the space of all possible three-dimensional parameter vectors, $\vec{\theta }=[\theta_{0},\theta_{1}^{A},\theta_{1}^{B}]$, for the all-order interaction model on sequences of length L=1 built from the alphabet {A,B}. Orange dot: initial choice of $\vec{\theta }=[0,\;0,\;2]$, which yields f(A)=0,f(B)=2. Red line: parameter vectors equivalent to $\vec{\theta }$. Blue plane: parameter vectors in the zero-sum gauge, i.e., that satisfy $\theta_{1}^{A}+\theta_{1}^{B}=0$. Green dot: gauge-fixed parameters ${\vec{\theta }}_{\text{fixed}}=[1,\;-1,\;1]$. (B,C) Algorithm performance when fixing the gauge of all-order and pairwise protein models with increasing number of parameters. Shown are the runtime (B) and peak memory (C) requirements for GaugeFixer and for standard matrix multiplication on a laptop computer. Error bars represent the standard deviation in runtime and memory usage over 10 projections of parameter vectors drawn i.i.d. from a normal distribution. (D) Sequence logos [Tareen and Kinney, 2019] showing the probability distributions π corresponding to different peaks in the Shine-Dalgarno fitness landscape measured by Kuo et al. [2020] and modeled byMartí-Gómez et al. [2026]. Each distribution has a fixed AGGAG motif at a specific register relative to the start codon. (E,F,G) Gauge-fixed constant (E), additive (F), and pairwise (G) parameters in the hierarchical gauge associated with each register. Horizontal dashed lines in (E) represent the average phenotype across all possible sequences (red) and the phenotype of the wild-type sequence AAGGAGGUG (green), which occurs in the 5’UTR of the *dmsC* gene of *Escherichia coli*. (H) Scatterplots comparing the values of gauge-fixed parameters observed in the core region at different registers.

To motivate the need for gauge fixing, consider a simple landscape over sequences of length L=1 built using a two-character alphabet {A,B} and defined by the fitness values f(A)=0,f(B)=2. To model this landscape we adopt a generalized one-hot model having three parameters—a constant-effect parameter ($\theta_{0}$) and two additive parameters ($\theta_{1}^{A}$ and $\theta_{1}^{B}$) that describe the effect of either character at position 1 in the sequence. One choice of parameters that describes the landscape is $\vec{\theta }=[\theta_{0},\theta_{1}^{A},\theta_{1}^{B}]=[0,\;0,\;2]$ (Figure 1A, orange dot). There are, however, an infinite number of equivalent parameter vectors we can choose, namely vectors of the form [z,-z,2-z] where z is any real number (red line). Fixing the gauge involves specifying an additional constraint that selects a single point on this line and thus specifies a unique choice of parameter values. The blue plane in Figure 1A represents one such constraint: the “zero-sum gauge,” which requires that the additive parameters sum to zero at each position. In the present example this restricts parameters to the plane defined by $\theta_{1}^{A}+\theta_{1}^{B}=0$. The resulting gauge-fixed parameters lie at the intersection of the line of equivalent parameters and the zero-sum plane (${\vec{\theta }}_{\text{fixed}}=[1,-1,1]$; green dot). Note that no choice of parameters in the zero-sum gauge other than ${\vec{\theta }}_{\text{fixed}}$ will yield the same landscape.

### Families of gauges

In previous work [Posfai et al., 2025b], we introduced a family of gauges for the all-order interaction model. These gauges are parameterized by two quantities: a non-negative number λ and a probability distribution π over sequences. λ controls how much explanatory power is allocated across interaction orders, while π specifies the distribution of sequences against which parameter effects are expressed. This λ,π family of gauges has multiple attractive properties. In particular, it encompasses the most commonly used gauges in the literature: these include the trivial gauge, the Euclidean gauge, the equitable gauge, the zero-sum gauge, and the wild-type gauge. See Posfai et al. [2025b] and SI Sec. 3 for details.

A particularly useful subset of gauges in this family are the hierarchical gauges, which are obtained in the limit where λ approaches infinity. In these gauges, lower-order terms explain as much of the landscape’s variance as possible, while higher-order interaction terms capture only residual variation that cannot be attributed to lower-order effects. Unlike other gauges in the λ,π family, hierarchical gauges can be applied to a much larger class of models called the “hierarchical models.” The values of model parameters in hierarchical gauges also have a natural interpretation: each parameter represents the average effect of introducing specific characters at specific positions, compared to the effect expected from lower-order terms, when sequences are drawn from the distribution π. By choosing different distributions π, one can observe how sequence-function relationships vary across different regions of sequence space.

## Results

### The GaugeFixer algorithm

Fixing the gauge requires projecting parameter vectors onto lower-dimensional subspaces, a computation performed by multiplying the parameter vector by a projection matrix. But when models have more than about 10^4^ parameters, such direct matrix multiplication becomes impractical owing to the need to generate and apply large projection matrices (which are not sparse). To overcome this limitation for all-order models, GaugeFixer exploits the fact that the projection matrices for all-order models can be written as Kronecker products of L much smaller matrices, one matrix for each sequence position [Posfai et al., 2025b]. This allows projections to be computed without ever constructing the full projection matrix [Martí-Gómez et al., 2026], thus reducing both the memory requirements and computation time from $O\left(M^{2}\right)$ to O(M) where M is the number of parameters. For the more general class of hierarchical models, GaugeFixer decomposes the model into a sum of all-order models restricted to subsets of positions, applies the efficient projection algorithm to each, and sums the results together (SI Secs. 4–5). Using these strategies, GaugeFixer achieves orders of magnitude improvement in both runtime and memory usage compared to gauge-fixing by direct matrix multiplication (Figures 1B,C). This dramatic improvement enables gauge-fixing computations to be performed on models with millions of parameters in just a few seconds on a standard laptop computer.

### Analysis of the Shine-Dalgarno fitness landscape

To illustrate the utility of GaugeFixer, we analyzed a fitness landscape for the Shine-Dalgarno (SD) sequence, a motif in bacterial messenger RNA that facilitates translation initiation by base-pairing with the 3’ tail of the 16S ribosomal RNA [Shine and Dalgarno, 1975]. Based on measurements of the translational activity of nearly every possible 9-nucleotide RNA sequence [Kuo et al., 2020], we previously inferred an all-order model having 1,953,125 parameters. The resulting fitness landscape contains multiple prominent fitness peaks corresponding to the canonical AGGAG motif positioned in different registers relative to the start codon [Zhou et al., 2022,Martí-Gómez et al., 2026].

Here, we used GaugeFixer to quantitatively characterize the local structure around the fitness peaks of this model. For each peak we defined a distribution π that fixes the AGGAG core motif in the corresponding register while randomizing the characters at the remaining positions (Figure 1D). We then imposed the hierarchical gauge corresponding to each choice of π and examined the resulting parameter values. In this gauge, the constant, additive, and pairwise parameters can be respectively interpreted as the average phenotype, average single-nucleotide effect, and average epistatic effect observed when mutations are introduced into sequences randomly drawn from π. We emphasize that these different parameter values, corresponding to different gauges, are simply different representations of the same model.

The constant term, $\theta_{0}$, represents the mean fitness of sequences with AGGAG in the specified register. We find that this value is highest for registers −12 and −11, consistent with known optimal spacing requirements for translation initiation (Figure 1E). In register −9, by contrast, the mean fitness is much lower, indicating markedly lower translation on average. The additive parameters in the core region, shown in Figure 1F, reveal how individual nucleotide mutations away from the AGGAG motif affect translational efficiency in each register. As expected, these mutations are overwhelmingly deleterious. The effects are also remarkably consistent across registers, though some differences emerge near the boundaries. The pairwise interaction parameters in the core region, shown in Figure 1G, capture the effects of mutating pairs of nucleotides within the AGGAG motif beyond what additive effects alone predict. These interactions are also remarkably consistent across registers. Predominantly positive values are observed, indicating that combinations of mutations tend to be less deleterious than expected from their individual effects, one hallmark of global epistasis [Otwinowski et al., 2018, Kinney and McCandlish, 2019].

Finally, we compared the constant, additive, and pairwise parameters in the core region across registers (Figure 1H). The results show that neighboring registers tend to have more similar parameters, while more distant registers show greater divergence. This smooth variation suggests that ribosomal binding preferences change gradually as a function of distance from the start codon.

## Discussion

Here we introduced GaugeFixer, a Python package that efficiently removes gauge freedoms from generalized one-hot models that describe sequence-function relationships. By exploiting the Kronecker factorization of projection operators and the specific mathematical structure of the models, GaugeFixer dramatically reduces runtime and memory requirements, thus enabling rapid gauge fixing for models with millions of parameters.

We wish to emphasize that gauge fixing is fundamentally different from parameter inference. Both procedures are an essential part of modeling sequence-function relationships, but they serve orthogonal purposes. Inference is the process of identifying parameters that make a model’s predictions best fit one’s data, but without regard to parameter interpretation. Gauge fixing, by contrast, alters model parameters in ways that have no effect on model predictions, but which are important for the interpretation of those parameters. GaugeFixer makes this distinction explicit by providing utilities to convert precomputed parameter vectors into any chosen gauge. A related but more subtle point is that some regularization schemes and/or prior distributions used during inference yield parameters in specific gauges [Posfai et al., 2025b, Petti et al., 2025]. This does not, however, eliminate the need for post-inference gauge fixing if model parameters are to be interpreted.

Different gauges have different advantages when interpreting model parameters. For instance, the influence of global epistasis is more evident when parameters are represented in the wild-type gauge, whereas using additive models to illustrate the specificities of transcription factors, or computing contact maps in pairwise interaction models, typically requires the models to be in the zero-sum gauge [Kinney et al., 2007, Stormo, 2011, Rube et al., 2022, Marks et al., 2011, Ekeberg et al., 2013]. Hierarchical gauges, on the other hand, facilitate the investigation of how mutational effects and their interactions change across different regions of sequence space, providing an intuitive way to interpret higher-order interactions.

Although GaugeFixer is designed to work with specific classes of linear models (all-order models and hierarchical models), it can also be applied to nonlinear and nonparametric models, such as neural networks or Gaussian processes, by representing the predicted landscape using an all-order model and applying GaugeFixer to the parameters of that model. While GaugeFixer requires exhaustive enumeration of all model parameters, recent theoretical results suggest the possibility of directly computing the posterior distribution over specific gauge-fixed parameters using Gaussian process models, thus bypassing the need to construct an equivalent all-order model with an astronomically large number of parameters [Petti et al., 2025]. Gauge fixing is also conceptually adjacent to neural network interpretability methods such as “global importance analysis” [Koo et al., 2021] or “in silico marginalization” [Schreiber, 2025, Liu et al., 2025].

GaugeFixer thus provides a high-performance software library for fixing the gauge of commonly used sequence-to-function models, thereby filling an important gap in the computational tools available for interpreting sequence-function relationships.

## Supplementary Material

Supplement 1

## Data Availability

GaugeFixer is compatible with Python ≥ 3.10 and can be installed using the pip package manager. Documentation is provided at https://gaugefixer.readthedocs.io. Source code is available at https://github.com/jbkinney/gaugefixer, as are the scripts used to carry out the analyses presented here.
