# Supplementary material for: GaugeFixer: overcoming parameter non-identifiability in models of sequence-function relationships
Source: bioRxiv. 2026 Feb 25:2025.12.08.693054. Originally published 2025 Dec 10. Preprint. [Version 2] doi: 10.64898/2025.12.08.693054 (PMC12713665; doi:10.64898/2025.12.08.693054)
Supplement: Supplementary file 1 [file media-1.pdf]

GaugeFixer: overcoming parameter non-identifiability in models of sequence-function relationships

Supplementary Information

Carlos Martí-Gómez[1], David M. McCandlish[1], and Justin B. Kinney[*1]

[1]Simons Center for Quantitative Biology, Cold Spring Harbor Laboratory, 1 Bungtown Rd., Cold Spring Harbor, NY 11724, United States

1 Generalized one-hot models

We consider scalar-valued sequence-function relationships $f(s)$ where s is a sequence of fixed length L built from an alphabet $\mathcal{A} = \{c_1, c_2, \ldots, c_\alpha\}$ comprising α distinct characters.[1] Let $S = \{1, 2, \ldots, L\}$ denote the set of positions within s. Linear models of sequence-function relationships have the form

$$f(s) = \vec{\theta} \cdot \vec{x}(s), \tag{1}$$

where $\vec{\theta}$ is a vector of model parameters and $\vec{x}(s)$ is a vector of sequence-dependent features.

This work focuses on a specific class of linear models, the generalized one-hot models described by Posfai et al. (2025b,a). In these models, the sequence-dependent features are binary indicator functions over subsequences. Formally, each feature is given by

$$x_U^u(s) = \begin{cases} 1 & \text{if } s[U] = u \\ 0 & \text{otherwise} \end{cases}, \tag{2}$$

where $U \subseteq S$ is a subset of positions, $s[U]$ indicates the subsequence of s that occurs at these positions, and u is a sequence of length $|U|$ (i.e., $u \in \mathcal{A}^{|U|}$).[2] Generalized one-hot models also require that $\vec{x}(s)$ be equivariant under position-specific character permutations (Posfai et al., 2025a). This means that if x_U^u occurs in $\vec{x}$ for some U and subsequence u, so must $x_U^{u'}$ for every possible subsequence u' of size $|U|$. The set of features in a generalized one-hot model is therefore defined by an alphabet $\mathcal{A}$ and a collection of position sets $V = \{U_1, U_2, \ldots, U_n\}$ via

$$f(s) = \sum_{U \in V} \sum_{u \in \mathcal{A}^{|U|}} \theta_U^u x_U^u(s). \tag{3}$$

*Corresponding author: jkinney@cshl.edu

[1]GaugeFixer actually supports sequences built using different alphabets at different positions, with no need for these alphabets to be of the same size. The formulas in this section generalize in a straightforward way to accommodate this more general use case.

[2]Sets of positions are called "orbits" in (Posfai et al., 2025b,a).

2 All-order models and hierarchical models

All-order models are generalized one-hot models that include features for all possible subsequences at all possible sets of positions. This means that V includes every possible position set $U \subseteq S$. V is therefore the powerset of S, denoted $V = \mathcal{P}(S)$. Because the subsets of S fully define V, we say that S is the generating position set of V.

Hierarchical models are more general than all-order models. Each hierarchical model is defined by one or more generating position sets $S' \subseteq S$, the subsets of which together comprise V. Formally, if $W = \{S'_1, S'_2, \ldots, S'_m\}$ denotes the set of generating position sets, then $V = \bigcup_{S' \in W} \mathcal{P}(S')$ is the union of the powersets of these generating position sets. Common examples of hierarchical models include additive models (defined by all position sets of size 0 and 1), pairwise models (all position sets up to size 2), nearest-neighbor models (same as pairwise but with size-2 position sets restricted to adjacent positions), K-order models (all position sets up to size K), and K-adjacent models (all position sets up to size K but restricted to K adjacent positions).[3]

3 Gauge-fixing projection matrices

The λ, π family is a set of gauges for the all-order interaction model. It is parameterized by a non-negative number λ and a probability distribution π over sequences that factorizes over positions. This family includes many commonly used gauges. In particular, the hierarchical gauges correspond to setting $\lambda = \infty$ and generalize two commonly used gauges. The zero-sum gauge, commonly used when modeling transcription factor binding to DNA, is the hierarchical gauge that results from π being uniform. Similarly, defining π to be an indicator function for a specific sequence yields the wild-type gauge, which is commonly used in studies of protein fitness landscapes.

Fixing the gauge of all-order models is accomplished by linear projection. In this case there are $M = (\alpha + 1)^L$ parameters. Given an M-dimensional vector $\vec{\theta}$ of initial parameter values, one can obtain the gauge-fixed parameters $\vec{\theta}_{\text{fixed}}$ via multiplication with an appropriate $M \times M$ projection matrix P (which depends on λ and π):

$$\vec{\theta}_{\text{fixed}} = P\vec{\theta}. \tag{4}$$

Importantly, the projection matrix can be written as a Kronecker product of position-specific matrices:

$$P = P_1 \otimes P_2 \otimes \cdots \otimes P_L, \tag{5}$$

where each P_l is the $(\alpha + 1) \times (\alpha + 1)$ matrix

$$P_l = \begin{pmatrix} \eta & \pi_l^{c_1}\eta & \pi_l^{c_2}\eta & \cdots & \pi_l^{c_\alpha}\eta \\ 1-\eta & 1-\pi_l^{c_1}\eta & -\pi_l^{c_2}\eta & \cdots & -\pi_l^{c_\alpha}\eta \\ 1-\eta & -\pi_l^{c_1}\eta & 1-\pi_l^{c_2}\eta & \cdots & -\pi_l^{c_\alpha}\eta \\ \vdots & \vdots & \vdots & \ddots & \vdots \\ 1-\eta & -\pi_l^{c_1}\eta & -\pi_l^{c_2}\eta & \cdots & 1-\pi_l^{c_\alpha}\eta \end{pmatrix}, \tag{6}$$

π_l^c is the probability of character c occurring at position l, and $\eta = \lambda/(1 + \lambda)$. See Posfai et al. (2025b) for details.

[3] Posfai et al. (2025a) adopted a different definition of K-adjacent models, one that makes these models non-hierarchical. We favor the present definition, which is hierarchical and thus amenable to gauge fixing using hierarchical gauges.

Although the λ, π gauges are defined in the context of all-order models, the specific class of hierarchical gauges can also be applied to the more general class of hierarchical models. This is done by treating each hierarchical model on sequences of length L as an all-order model on sequences of the same length that has unused parameters set to zero. This works because hierarchical gauges preserve these sets of zero-valued parameters. Other λ, π gauges do not preserve these zero-valued parameters, and therefore are restricted in their application to all-order models only.

4 Efficient gauge fixing via Kronecker factorization

To compute $P\vec{\theta}$, we first reshape $\vec{\theta}$ to be an L-dimensional tensor having size $(\alpha+1)$ along each dimension. The expression in Eq. 4 then becomes

$$[\vec{\theta}_{\text{fixed}}]_{i_1 \cdots i_L} = \sum_{j_1 \cdots j_L} [P_1]_{i_1 j_1} \cdots [P_L]_{i_L j_L} [\vec{\theta}]_{j_1 \cdots j_L}. \tag{7}$$

We compute Eq. 7 iteratively: first we define $\vec{\theta}^{(0)} = \vec{\theta}$; then for $l = 1, 2, \cdots, L$ we compute

$$\vec{\theta}^{(l)}_{i_1 \cdots i_L} = \sum_{j_l} [P_l]_{i_l j_l} [\vec{\theta}^{(l-1)}]_{i_1 \cdots i_{l-1} j_l i_{l+1} \cdots i_L}, \tag{8}$$

which ultimately yields $\vec{\theta}_{\text{fixed}} = \vec{\theta}^{(L)}$. This computation is formalized in Algorithm 1, which uses a single intermediate parameter vector $\vec{\psi}$ to avoid storing all $\vec{\theta}^{(l)}$ simultaneously. Algorithm 1 has a computational complexity of $\mathrm{O}(L(\alpha+1)M)$. This compares favorably to the computational complexity of direct matrix multiplication of $\vec{\theta}$ by P, which is $\mathrm{O}(M^2)$. The memory requirements of Algorithm 1 also scale linearly with M, as opposed to quadratically with M for direct matrix multiplication.

Algorithm 1 Fixing the gauge of all-order models

Require: $\lambda, \pi, \vec{\theta}_{\text{init}}$
$\vec{\psi} \leftarrow \text{reshape}(\vec{\theta}_{\text{init}})$
for $l = 1$ to L **do**
  $P_l \leftarrow \text{ProjectionMatrix}(\lambda, \pi_l)$ ▷ Equation 6
  $\vec{\psi} \leftarrow \text{tensordot}(P_l, \vec{\psi}, l)$ ▷ Equation 8
end for
$\vec{\theta}_{\text{fixed}} \leftarrow \text{reshape}(\vec{\psi})$
return $\vec{\theta}_{\text{fixed}}$

5 Hierarchical model decomposition

To project a hierarchical model into a hierarchical gauge, we leverage the fact that the parameters of the model can be expressed as a sum of the parameters of multiple all-order models, each restricted to one of the generating position sets. Let $\vec{\theta}$ be the parameter vector of the hierarchical model expressed as an $(\alpha+1)^L$-dimensional all-order parameter vector (with unused parameters set to zero), and let W denote the set of generating position sets. We first decompose $\vec{\theta}$ as

$$\vec{\theta} = \sum_{S' \in W} \vec{\theta}_{S'}, \tag{9}$$

where each $\vec{\theta}_{S'}$ is an all-order parameter vector for the position set S', i.e., has nonzero parameters θ^u_U only for $U \subseteq S'$. We note that there are multiple ways to carry out this decomposition, all of which yield the same gauge-fixed parameters. The gauge-fixed parameters are then computed as

$$\vec{\theta}_{\text{fixed}} = \sum_{S' \in W} P \vec{\theta}_{S'}. \tag{10}$$

Importantly, each term on the right-hand side can be computed using only the Kronecker factors P_l corresponding to positions $l \in S'$. Thus, there is never any need to represent the full $(\alpha+1)^L$-dimensional vectors $\vec{\theta}$, $\vec{\theta}_{S'}$, or $\vec{\theta}_{\text{fixed}}$.

Algorithm 2 formalizes this procedure. Here, $\vec{\theta}[S']$ indicates an $(\alpha+1)^{|S'|}$-dimensional vector composed only of the elements θ^u_U for which $U \subseteq S'$, and $\pi[S']$ represents the marginal distribution π over subsequences on S'. Note that the iterative form of this algorithm allows us to avoid simultaneously storing the nonzero elements of all $\vec{\theta}_{S'}$ vectors.

Algorithm 2 Fixing the gauge of hierarchical models.

Require: $\pi, \vec{\theta}_{\text{init}}, W$
$\vec{\theta}_{\text{fixed}} \leftarrow \vec{0}$
$\vec{\theta}_{\text{tmp}} \leftarrow \vec{\theta}_{\text{init}}$
for S' in W **do**
  $\vec{\omega} \leftarrow \vec{\theta}_{\text{tmp}}[S']$ ▷ Note: $\vec{\omega} = \vec{\theta}_{S'}[S']$
  $\vec{\omega}_{\text{fixed}} \leftarrow \text{FixAllOrders}(\infty, \pi[S'], \vec{\omega})$ ▷ Algorithm 1
  $\vec{\theta}_{\text{fixed}}[S'] \leftarrow \vec{\theta}_{\text{fixed}}[S'] + \vec{\omega}_{\text{fixed}}$
  $\vec{\theta}_{\text{tmp}}[S'] \leftarrow \vec{0}$
end for
return $\vec{\theta}_{\text{fixed}}$

References

Anna Posfai, David M. McCandlish, and Justin B. Kinney. Symmetry, gauge freedoms, and the interpretability of sequence-function relationships. *Physical Review Research*, 7(2):023005, 2025a. doi: 10.1103/physrevresearch.7.023005.

Anna Posfai, Juannan Zhou, David M. McCandlish, and Justin B. Kinney. Gauge fixing for sequence-function relationships. *PLOS Computational Biology*, 21(3):e1012818, 2025b. ISSN 1553-734X. doi: 10.1371/journal.pcbi.1012818.
